# Bone marrow cell extract promotes the regeneration of irradiated bone

**DOI:** 10.1371/journal.pone.0178060

**Published:** 2017-05-18

**Authors:** Guillaume Michel, Pauline Blery, Michaël Henoux, Jérôme Guicheux, Pierre Weiss, Sophie Brouard, Olivier Malard, Florent Espitalier

**Affiliations:** 1 Service d'O.R.L. et de chirurgie cervico-faciale, Centre Hospitalier Universitaire, Nantes, France; 2 INSERM, UMRS 791, LIOAD, Université de Nantes, Nantes, France; 3 Service d’Odontologie Restauratrice et Chirurgicale, Centre Hospitalier Universitaire, Nantes, France; 4 INSERM UMR 1064, ITUN, Université de Nantes, Nantes, France; Centro Cardiologico Monzino, ITALY

## Abstract

Mandibular osteoradionecrosis is a severe side effect of radiotherapy after the treatment of squamous cell carcinomas of the upper aerodigestive tract. As an alternative to its treatment by micro-anastomosed free-flaps, preclinical tissular engineering studies have been developed. Total bone marrow (TBM) associated with biphasic calcium phosphate (BCP) significantly enhanced bone formation in irradiated bone. One mechanism, explaining how bone marrow cells can help regenerate tissues like this, is the paracrine effect. The bone marrow cell extract (BMCE) makes use of this paracrine mechanism by keeping only the soluble factors such as growth factors and cytokines. It has provided significant results in repairing various tissues, but has not yet been studied in irradiated bone reconstruction. The purpose of this study was to evaluate the effect of BMCE via an intraosseous or intravenous delivery, with a calcium phosphate scaffold, in irradiated bone reconstruction.

Twenty rats were irradiated on their hind limbs with a single 80-Gy dose. Three weeks later, surgery was performed to create osseous defects. The intraosseous group (*n* = 12) studied the effect of BMCE in situ, with six combinations (empty defect, BCP, TBM, BCP-TBM, lysate only, BCP-lysate). After four different combinations of implantation (empty defect, BCP, TBM, BCP-TBM), the intravenous group (*n* = 8) received four intravenous injections of BMCE for 2 weeks. Five weeks after implantation, samples were explanted for histological and scanning electron microscopy analysis. Lysate immunogenicity was studied with various mixed lymphocyte reactions.

Intravenous injections of BMCE led to a significant new bone formation compared to the intraosseous group. The BCP-TBM mixture remained the most effective in the intraosseous group. However, intravenous injections were more effective, with TBM placed in the defect, with or without biomaterials. Histologically, highly cellularized bone marrow was observed in the defects after intravenous injections, and not after an in situ use of the lysate. The mixed lymphocyte reactions did not show any proliferation after 3, 5, or 7 days of lysate incubation with lymphocytes from another species.

This study evaluated the role of BMCE in irradiated bone reconstruction. There were significant results arguing in favor of BMCE intravenous injections. This could open new perspectives to irradiated bone reconstruction.

## Introduction

Treatment of squamous cell carcinomas of the upper aerodigestive tract remains a major health challenge today, with more than 263,000 new cases per year in the world [1]. Their treatment often requires surgery and postoperative high-dose external radiotherapy. Both have major side effects. Surgery requires large removal and induces long-term esthetic and functional disorders. Radiotherapy reduces healing capacities because of the decrease of bone vascularization [2]. Mandibular osteoradionecrosis (ORN) is a severe side effect of radiotherapy that affects 5% of treated patients [3] despite preventive actions. It leads to mandibular fractures and deglutition and phonation disorders [4]. The standard reconstruction procedure for extended ORN is the micro-anastomosed free-flap [5]. However, this procedure requires prolonged general anesthesia, with a higher rate of complications on postradiation patients [6].

In this context, preclinical studies have been developed using calcium phosphate biomaterials. Total bone marrow (TBM) associated with biphasic calcium phosphate (BCP) significantly enhanced bone formation in irradiated bone [7][8]. One of the mechanisms explaining how bone marrow cells can help regenerate such tissues is the paracrine effect [9]. Bone marrow cells release soluble factors such as cytokines and growth factors that induce neovascularization, cytoprotection and tissue regeneration [10]. To study this paracrine effect, a number of recent studies have developed a bone marrow cell extract (BMCE) containing intracellular factors. Thus, in infarcted heart [11][12] as in irradiated salivary glands [13], injection of BMCE leads to an improvement comparable to intact cell therapy. Moreover, both intravenous and intraglandular injections are as effective in repairing irradiated salivary glands [13].

BMCE has not been studied in irradiated bone reconstruction. However, it could repair the tissues damaged by radiotherapy by increasing neovascularization and tissue remodeling [10]. The purpose of this study was to evaluate the role of BMCE and the calcium phosphate scaffold to promote bone formation after irradiation. Intravenous and intraosseous injections with different combinations were compared to the BCP-TBM association, currently the most efficient material available.

## Materials and methods

### 1. Biphasic calcium phosphate

The biomaterial used for this study was granules of a macroporous biphasic calcium phosphate (MBCP^™^, Biomatlante, Vigneux de Bretagne, France). The granules were about 800 μm in diameter and were composed of hydroxyapatite and β tricalcium phosphate in a 60/40 ratio corresponding to a 1:60 ratio of Ca:P. The measured mean porosity was 40 ± 10%. Eppendorf tubes (Costar, Corning, NY, USA), each containing 0.015 g of MBCP^*™*^, were steam-sterilized at 121°C for 20 min before implantation.

### 2. Animals

**Fig 1:**
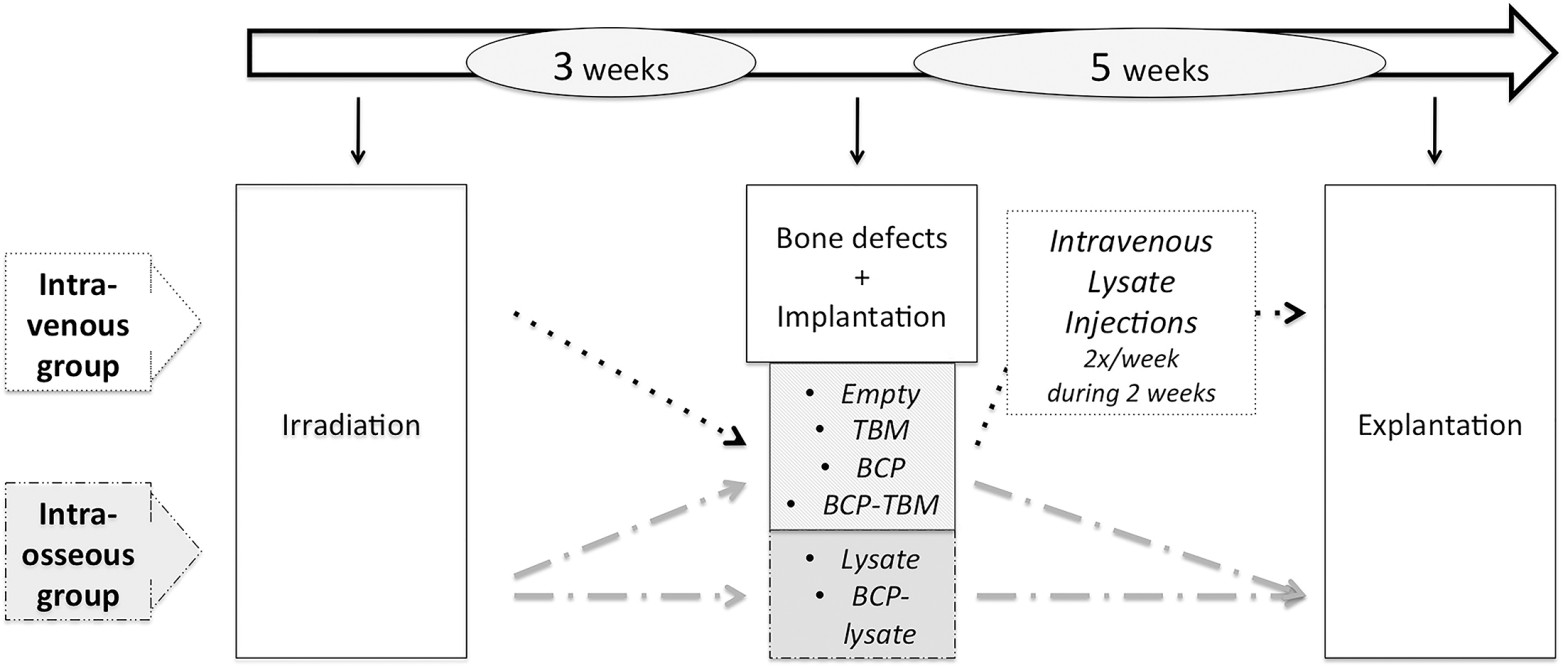
Outline of the protocol. Events concerning all animals (*n* = 20): white; events for the IV group (*n* = 8): dotted; events for the intraosseous group (*n* = 12): dashed.

The study was conducted on 8-week-old inbred male Lewis 1A-haplotype RT1^a^ rats (*n* = 27) provided by a certified breeding center (R. Janvier, Le Genest St. Isle, France), weighing >225 g. Animal care was provided in accordance with European directive number DE2010/063EU, after the “Ethics Committee for Animal Experimentation of Pays de la Loire” approval and Ministry of Research validation n°1315. Animal euthanasia was performed with an intracardiac injection of 50 mg of thiopenthal. Animals were examined clinically every day, including food intake and walking ability. A weighing was carried out once a week. Pain was managed by immediate post operative subcutaneous injection of meloxicam (1 mg/kg) then mixed with water during five days. The outline of the whole protocol is presented in Fig 1. Seven rats were specially designated as donors: two TBM donors and five BMCE donors. Twenty rats underwent external irradiation. Bone defects and implantations were performed 3 weeks after irradiation. The “intraosseous group” (*n* = 12) received injections of BMCE in the bone defect, with different combinations. The “intravenous group” (*n* = 8) received intravenous injections of BMCE as an adjuvant to the bone implantations. Five weeks after implantations, i.e., 8 weeks after irradiation, implanted bones were removed immediately after euthanasia.

### 3. Radiation delivery procedure

The radiation delivery was performed at the Institut de Cancérologie de l’Ouest (Centre de Lutte Contre le Cancer René Gauducheau, Saint Herblain, France). Twenty rats were irradiated under general anesthesia induced by an isoflurane inhalation (Forène^®^, Abbott France, Rungis, France). Then the animals were placed in the feet-first prone position in the XRAD225Cx irradiator (Precision X-Ray Inc., North Branford, CT, USA). A single 80-Gy dose was administered on their hind limbs. The purpose was to reproduce the histological and mechanical effects of irradiation, appearing beyond 70 Gy [14]. The target was the patella, with half of the femoral height and half of the tibial height in the radiation field. The field diameter was 2.8 cm at the isocenter. A total of 572 s per foot were needed to obtain the required dose of 80 Gy on the cortical bone, at 225 kVp and an intensity of 13 mA. The absorbed dose rate on the cortical bone was 8.39 Gy/min, and 3.3 Gy/min on the surrounding soft tissues.

An absorbed dose calculation was made using Monte Carlo simulation [15], based on CT images of the first irradiated rat (voxels, 0.2×0.2×0.2 mm^3^), with a statistical dose variation below 4% in the targeted bone and below 8% in the adjacent soft tissue.

### 4. Surgical procedure

All the surgical procedures were performed under general anesthesia using 4% isoflurane inhalation for induction and 2% for preservation.

#### 4.1. Isolation of TBM

The TBM was collected the day of the implantations from femoral, tibial, and humeral bones of two nonirradiated donors. The extremities of each bone were cut and 6 mL of physiological saline PBS (Gibco, 1X, pH 7.2) was infused through the medullar cavity. The TBM was collected and centrifuged (5 min at 600 *g*). The cell pellet was resuspended in physiological saline and a nucleated cell count was performed using both the trypan blue exclusion test and cytological myelographic analysis. The TBM was immediately placed in tubes containing anticoagulant (Vacuette Premium^®^, Greiner bio-one, Germany). An average of 10^7^ cells were implanted in each defect filled with TBM.

#### 4.2. BMCE preparation

Five days before implantation, the tibial, femoral, and humeral bones of five nonirradiated donors were collected to prepare the BMCE according to a protocol previously used in other studies [11][12][13]. The TBM collection technique was the same as described in section 4.1. Then the suspension was strained through a 70-μm nylon filter and the cell concentration was adjusted to 10^7^ viable cells/μL. The cells were subjected to three freeze—thaw cycles, using a −80°C freezer and thawing in a 37°C bath, followed by microcentrifugation at 14,000 *g* at 26°C for 30 min (Centrifuge 5804R, Eppendorf, Germany) to remove insoluble materials. The total number of nucleated cells was 493 × 10^7^. Forty-eight injections of BMCE from 10^7^ cells were performed.

#### 4.3. Implantations

Surgery was performed as previously described [16]: 3-mm-diameter osseous defects were surgically created in bilateral tibial and femoral metaphysis (four defects per animal). The defects were then totally filled with either BCP only, TBM only, a mixture of BCP and TBM, or no filling.

The “intravenous group” received intravenous injections of extract obtained from 10^7^ cells in 0.5 mL physiological saline. The injections were performed in the dorsal vein of the penis, twice a week over 2 weeks under general anesthesia.

The defects of the “intraosseous group” were filled with two added mixtures: BMCE only (50 μL cell extract from 10^7^ cells) and a mixture of BCP and BMCE.

Postoperative analgesia was immediately supported by a subcutaneous injection of buprenorphine (0.1 mg, Buprécare^®^, Animalcare, York, Great Britain), repeated at day 3 and day 5, and by a subcutaneous injection of meloxicam (1 mg/kg, Metacam^®^, Boehringer, Germany), mixed with water over the following 5 days. An antibiotic treatment of marbofloxacin (3 mg/kg, Marbocyl^®^, Vétoquinol, Paris, France) was started the day of the surgery and repeated for 5 days.

#### 4.4. Histological examination

Five weeks after implantation, all the animals were sacrificed. Femurs and tibiae were then dissected and fixed for 5 days in a 4% paraformaldehyde phosphate-buffered saline. After dehydration through a graded series of ethanol and acetone, nondecalcified bone specimens were infiltrated and embedded in methyl-methacrylate resin, which hardens at low temperature (Technovit 9100, Kulzer, Wehrheim, Germany). After different preinfiltration and infiltration steps, the polymerization mixture was combined with bone samples and placed at −20°C for 10 days. After polymerization, serial 5-μm sections were cut perpendicular to the osseous defects and the surrounding bone using a diamond saw made for nondecalcified tissues (Polycut SM2500, Leica, Wetzlar, Germany). The bone sections were then stained with Movat pentachrome using an automated slide stainer (Shandon Gemini ES, ThermoFisher Scientific, Waltham, USA) and observed with a digital slide scanner (NanoZoomer 2.0 HT, Hamamatsu Photonics, Hamamatsu, Japan).

#### 4.5. Scanning electron microscopy and quantitative image analysis

Each bone sample was sanded then gold-palladium-coated. Scanning electron microscopy (SEM) studies of implanted defects were conducted with backscattered electrons (Leo 1450 VP, Zeiss, Oberkochen, Germany) in conjunction with image analysis. The quantity of newly formed bone and ceramic degradation was determined using a semi-automatic image analyzer procedure from SEM observations (Leica Qwin Pro v3.5.1) as previously described [17]. The quantity of newly formed bone, soft tissue, and remaining BCP was automatically calculated within the total area.

#### 4.6. Immunogenicity

Mixed lymphocyte reactions (MLR) were performed in the Research Center in Transplantation and Immunology INSERM UMR 1064, Nantes, France. We collected total splenocytes and performed a cellular marking with CellTrace Violet (1 μL CTV for 10^6^ cells). A cell culture with a gradation of BMCE from 0 to 50 μL and 200 to 150 μL of total splenocytes in a culture medium (RPMI- 1% PS– 1% GLU– 10% SVF; Gibco) was performed at 37°C. BMCE from a Lewis 1A rat was incubated with total splenocytes from a Wistar rat, and vice versa. After 3, 5, and 7 days of incubation, a viability marking was made using 3.5 μL of Fixable Viable eFluor 660, then a flow cytometry analysis. For each condition, viable lymphocytes were selected and their CTV expression was measured to observe any cell proliferation.

#### 4.7. Statistical analysis

Bone ingrowth and BCP resorption were compared using the Mann-Whitney and Kruskal-Wallis tests with statistical significance set at 5% (*p*<0.05).

## Results

### 1. Radiation delivery and surgical procedures

No death occurred during this study. Both the radiation delivery and surgical procedure were well tolerated with daily care. Eight weeks after irradiation, 51 samples were included in this study. The other samples could not be interpreted, particularly because of fractures.

### 2. SEM and image analysis

#### 2.1. Qualitative SEM study

**Fig 2:**
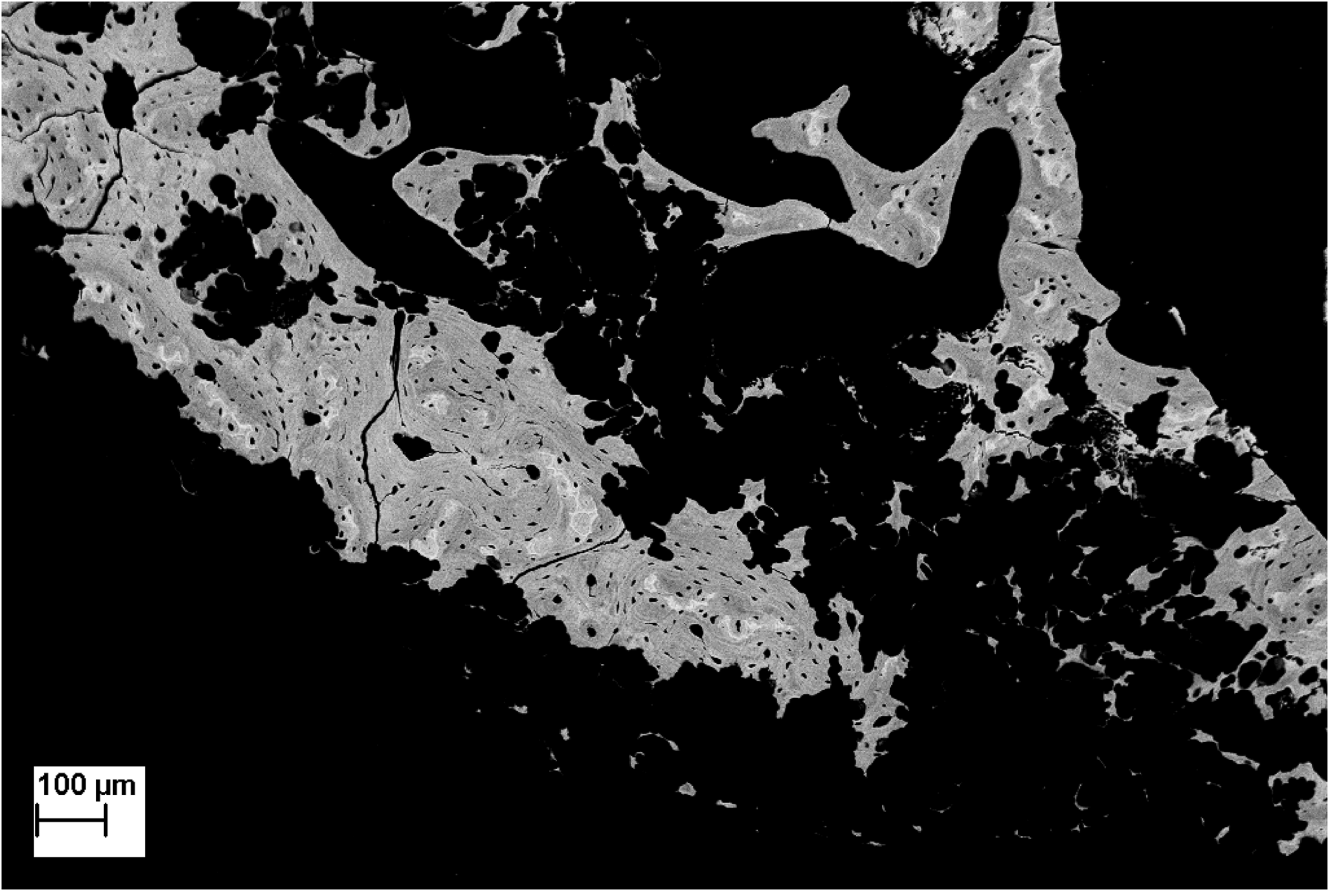
SEM backscattered electron image of irradiated bone (×60 magnification). There is destruction of the bone microarchitecture and presence of poly-lobed gaps, suggesting Dambrain periosteocytic lysis.

**Fig 3:**
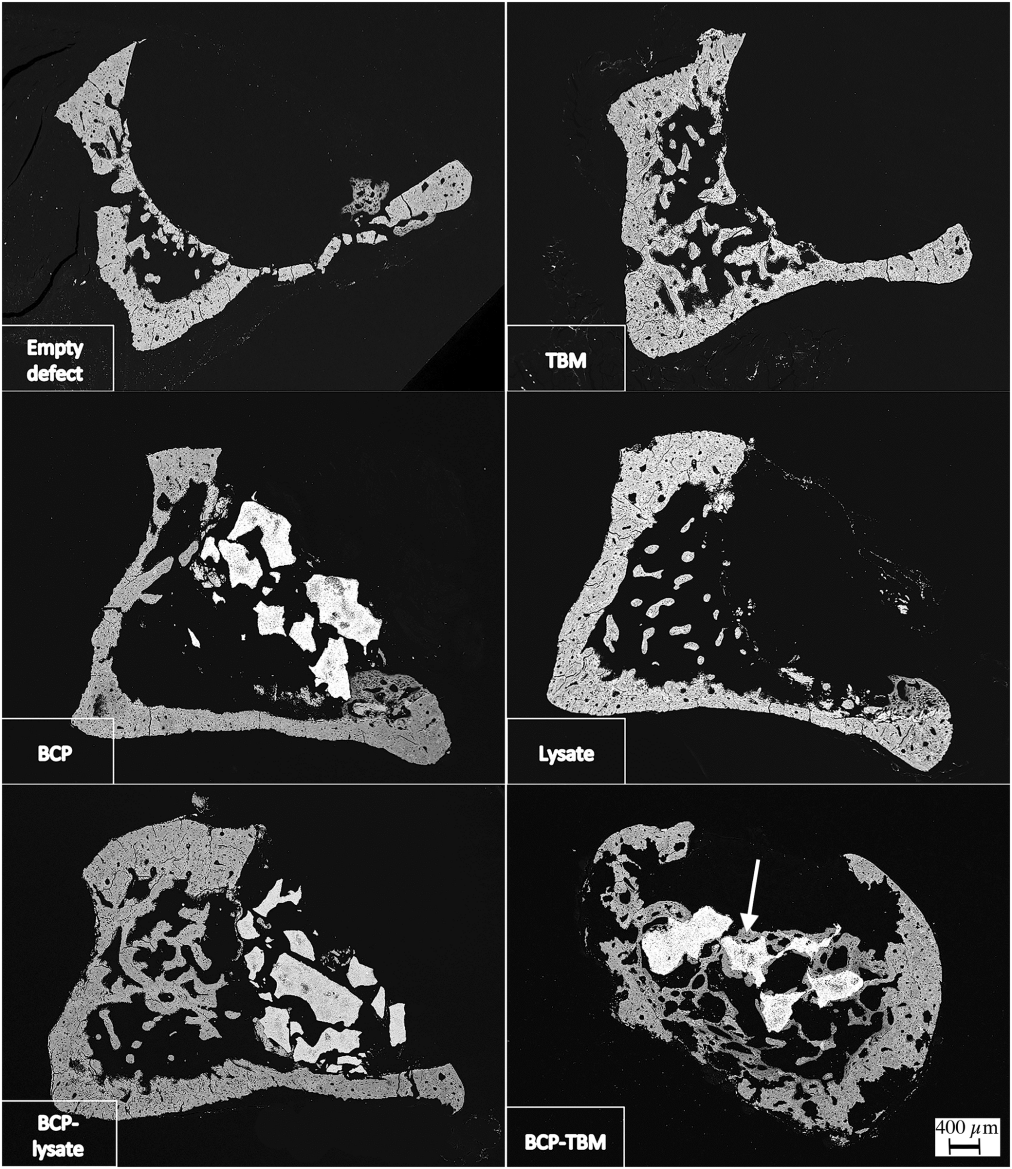
SEM backscattered electron images of samples explanted 8 weeks after irradiation from the intraosseous group (×20 magnification). Only the BCP-TBM mixture induces significant new bone formation. White arrow indicates new bone formation.

**Fig 4:**
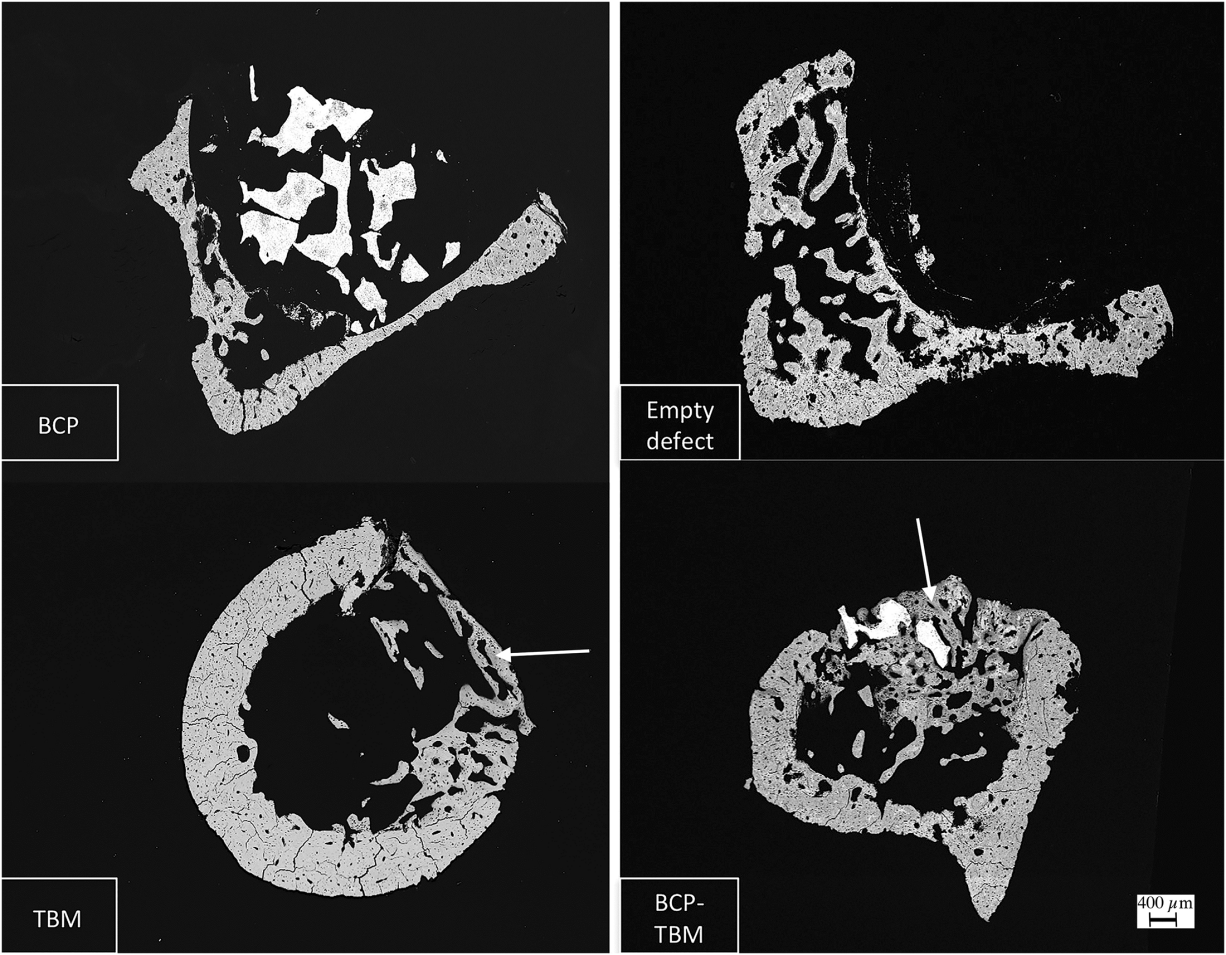
SEM backscattered electron images of samples from the IV group (×20). Defects filled with BCP-TBM and TBM only and followed by IV injections induced significant new bone formation. White arrows indicate new bone formation.

A highly necrotic bone with wide gaps [18] was observed in every sample (Fig 2). In the “intraosseous group,” no bone formation was observed except with the BCP-TBM mixture implantation (Fig 3). In the “intravenous group,” there was visible new bone formation on every sample filled with TBM or BCP-TBM (Fig 4).

#### 2.2. Quantitative SEM study

**Fig 5:**
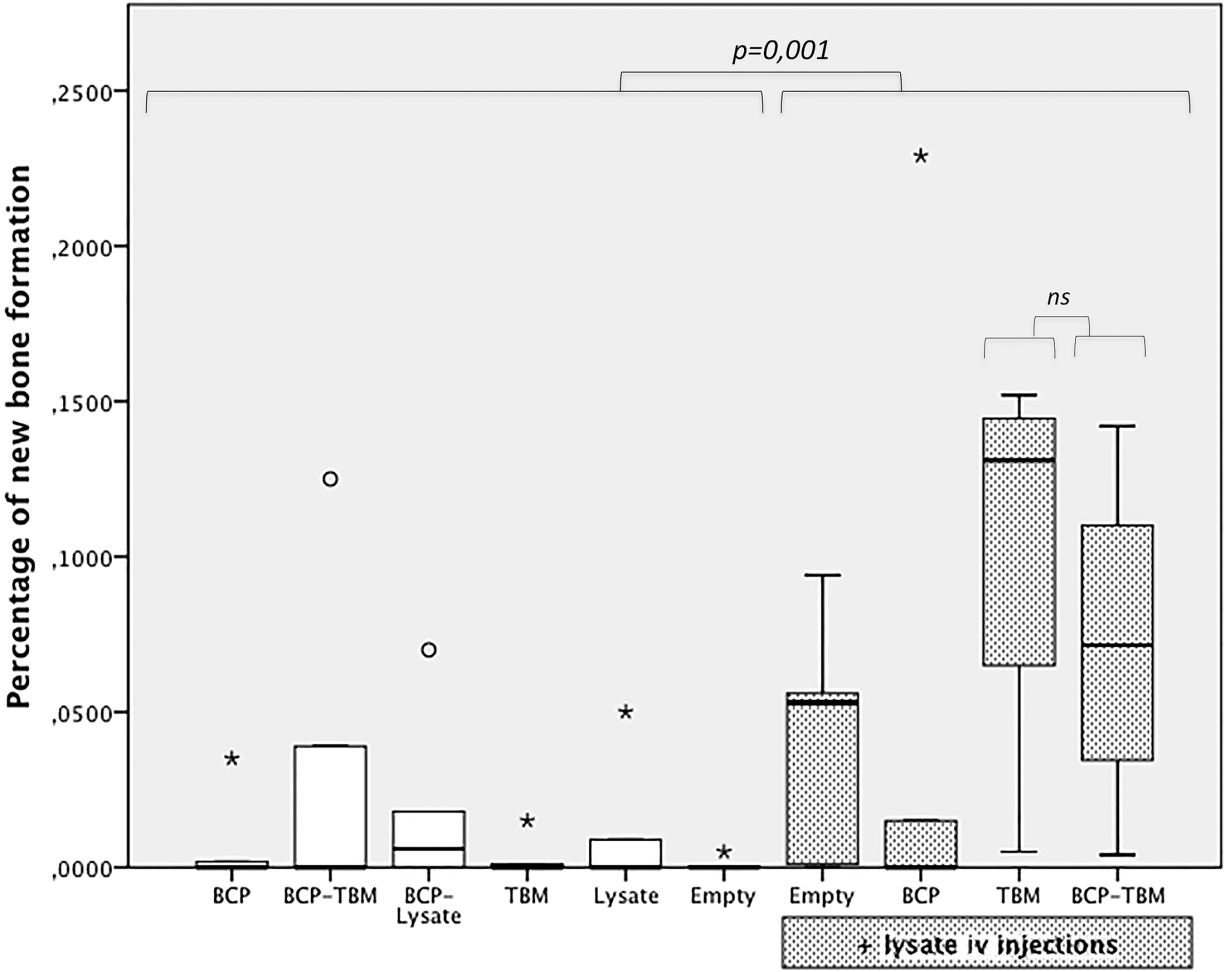
Box plots representing the percentage of newly formed bone according to the different combinations. The circles are outliers, and the asterisks are extreme outliers. The rate of bone ingrowth is significantly higher after lysate IV injection, particularly with TBM in the defect, with or without BCP (ns).

The ratio of bone ingrowth was significantly higher:

- in the “intravenous group” (BCP, TBM, empty defect, BCP-TBM) compared to the “intraosseous group” (BCP, TBM, empty defect, BCP-TBM) (*p* = 0.001) (Fig 5).
- after implantation of TBM followed by intravenous BMCE injections compared to the empty defect (*p* = 0.002), BCP alone (*p* = 0.007), TBM alone (*p* = 0.012), BMCE alone (*p* = 0.021), or with BCP (*p* = 0.008), BCP-TBM (*p* = 0.019), and BCP followed by intravenous BMCE injections (*p* = 0.044).
- after implantation of BCP-TBM followed by intravenous BMCE injections compared to the empty defect (*p* = 0.005), BCP alone (*p* = 0.018), TBM alone (*p* = 0.027), BMCE alone (*p* = 0.044) or with BCP (*p* = 0.018) or BCP-TBM (*p* = 0.041).

Finally, the ratio of bone ingrowth was significantly higher after TBM or BCP-TBM followed by intravenous BMCE injections than every other mixture (respectively, *p* = 0.003 and *p* = 0.012, Fig 5).

### 3. Histological examination

**Fig 6:**
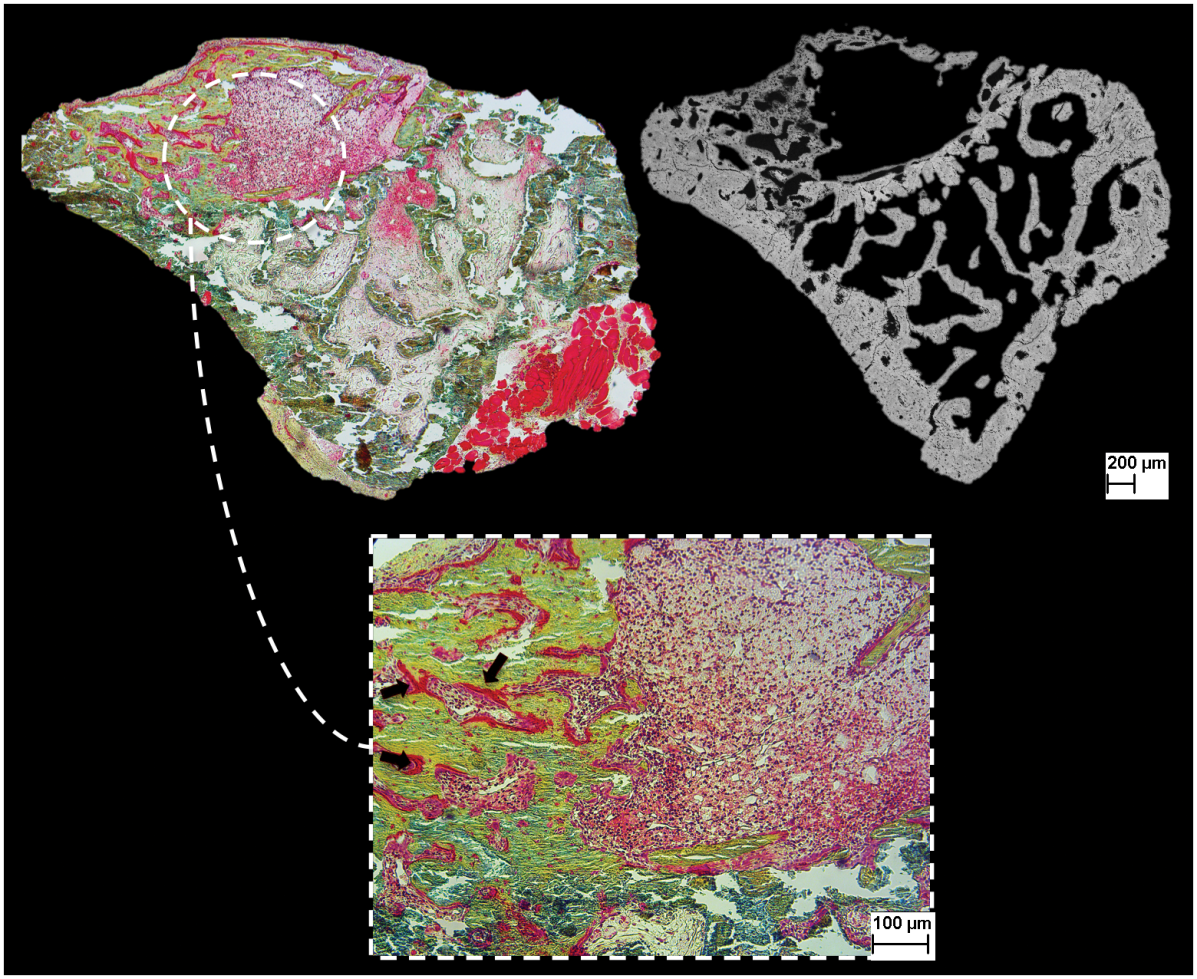
Image of histological analysis *(Movat pentachrome staining)* and correlation with SEM examination. Defect is filled with TBM, followed by IV injections. There is substantial bone formation, with numerous osteoid areas (black arrows) and rich bone marrow.

The aspect of newly formed bone, the connection between BCP and surrounding tissues and the formation of blood vessels in the osseous defects were evaluated. The histological examination was correlated with the qualitative SEM study (Fig 6).

After TBM or BCP-TBM implantation followed by intravenous BMCE injections, there was new bone formation towards the center of the defect. In both conditions, osteoblasts were arranged in rows to synthesize the osteoid matrix (data not shown). Newly formed bone was surrounded by many medullar cells. This highly cellularized bone marrow was observed in the defects after intravenous injections and not after an in situ use of the lysate. In the other associations, there was either no bone formation or new bone formation at the periphery of the defects. A noncellular tissue filled the defects, consistent with fibrosis.

### 4. Immunogenicity study

MLR analysis after 3, 5, and 7 incubation days did not show any lymphocyte proliferation, regardless of the condition used. The ratio of proliferative cells was always below 2%, and most of the time below 1%. Statistical analysis confirmed no difference between the MLR. Adding a BMCE from another species did not lead to a proliferation reaction.

## Discussion

Histological effects on bone were observed from single 20-Gy doses [19], with osteopenia. Osteopenia appeared as a consequence of a primary reduction of the number and the activity of osteoblasts [20], then an osteocyte reduction. Bone morphology and its biomechanical properties were degraded because of an alteration of the cellular pool and the vasculature, then progressive fibrosis and local necrosis [2]. We used an animal model that had been previously developed [16] to mimic the side effects of irradiation on bone. After histological examination, wide fibrosis areas and a decrease of medullar cells were observed. Numerous polycyclic gaps consistent with periosteocytic lysis, described by Dambrain as pathognomonic of ORN [18], were observed on SEM analysis. Furthermore, our radiation delivery procedure was reproducible and not deadly.

The BCP-TBM mixture was the most effective combination to induce new bone formation after irradiation [16]. Among the available biomaterials, BCP was chosen because of its biocompatibility, biodegradability, and osteoconductivity properties. TBM was included because of its regenerative capacity, due to various mechanisms: differentiation into osteoblastic lineage [21], vasculogenesis from endothelial progenitor cells contained in the bone marrow [22], and release of soluble factors acting in a paracrine fashion [10]. This is the mechanism we studied by lysing medullar cells to keep only the soluble factors. BMCE has previously been used in infarcted heart with a significant improvement of the left ventricular ejection fraction and a reduced infarct size [11]. The BMCE was as effective as whole live BM cells in repairing infarcted heart, suggesting a paracrine effect of MB cells. Other studies used the same protocol to prepare the BMCE and tested it in various tissues. On irradiated salivary glands [13], intraglandular or intravenous (IV) injections were both effective in restoring salivary flow rates to normal levels and increasing progenitor cells. Both BMCE delivery pathways were as effective as whole live BM cells. For salivary glands with Sjögren disease [23], as for erectile function recovery after cavernous nerve injury [24], significant results were obtained with BMCE injections.

This is, to our knowledge, the first study evaluating BMCE in repairing irradiated bone. We did not identify significant results on bone formation with in situ injections, unlike other previously studied tissues [11][13]. It is possible that the soluble factors contained in the lysate injected in bone did not find enough substrate to act, considering cellular depletion and hypovascularization [25]. A dog study did not find significant bone formation after in situ association of MSC lysate and β-TCP [26]. The fibrosis subsequent to ORN [27] could have limited the lysate circulation.

However, IV injections of BMCE enhanced bone formation with osseous implantation of BCP-TBM or TBM only. IV injections of BMCE, including all combinations, gave better results than the condition without IV injections. These injections were histologically associated with highly cellular bone marrow in bone defects. These results were observed despite the high levels of radiation used, far beyond the 15 Gy delivered to salivary glands [13], and despite the 3-week delay after irradiation to perform injections. This prolonged time frame has been chosen so as to be closer to clinical reality. In comparison, Tran et al [13] tested IV injections 5 days after irradiation, while repair capacity is not totally degraded. We highlighted new bone formation in an established ORN. Finally, considering intraosseous injections only in the present study, the BCP-TBM mixture still is, as published elsewhere [16], the best mixture to improve bone formation. However, IV injections led to a greater gain, in association with TBM in situ, with or without biomaterial.

To understand these results, proteins contained or stimulated by the lysate must be identified. A recent study [28] characterized the angiogenesis-related growth factors in BMCE, using a microarray approach. They identified nine angiogenesis-related proteins, like HGF and MMP8-9, and four cytokines. After deactivation of these factors, injections of deactivated BMCE was no better than injection of saline, while injections of native BMCE restored functions and vascularization of irradiated salivary glands. This study is a first step to understand results of BMCE injections, involving native proteins, but not the nucleic acids, lipids or carbohydrates.

This mechanism, using the secretome of TBM cells, is already known for MSCs. It has been proved that intracoronary injections of the MSC culture medium only led to a reduced infarct size in a pig model [29]. But BMCE includes all cell types of whole BM and consequently numerous proteins, cytokines, and paracrine factors [11]. For example, MMP8-9 and osteopontin are highly expressed in BMCE [28], and are known as factors degrading the matrix surrounding endothelial cells and promoting their migration, proliferation, then angiogenesis [30–32].

The highest factor expressed in BMCE was the Platelet Factor 4 (PF4) [28]. This factor is known to protect MSCs and bone marrow niches from irradiation, by reducing DNA damage and increasing survival of MSCs [33]. BMCE injections could counter the effects of radiotherapy on MSCs and their microenvironment.

Furthermore, we found that histologically, defects filled with TBM presented a high density of cells only after IV injections. It may be an action of SDF-1 and IL-1ra, found in BMCE [28]. SDF-1 is a factor inducing stem cells to migrate from the bone marrow to the damage site [34]. IL-1ra was reported to stimulate macrophages into a wound healing phenotype and promoting endothelial cell migration [35].

The BMCE is easy and quick to obtain. It does not involve the injection of live cells, which carry the risk of differentiating into tumorigenic cells [13], but it does involve potential systemic effects by IV injections, and therefore it must be considered only in the remission period, never with cancerous cells in place. We evaluated the possible immunogenicity of the BMCE. We performed several MLRs to determine whether lymphocyte proliferation appeared in the presence of a BMCE from another species. The lack of any proliferation argues in favor of a lack of immunogenicity. These results are concordant with the literature that has presented the BMCE as nonimmunogenic [12] [13] [23]. Studying the lack of immunogenicity, Angeli et al. [12] injected a human BMCE in a rat infarcted heart model. It improved heart function, without using immunosuppressed animals or immunosuppressive treatment, and without any sign of a graft-versus-host reaction. Fang et al [28] had similar findings, with a protein-mediated action of BMCE less tumorigenic and immunogenic than cell therapies. With no immune consequences, the lysate could be produced in advance from a human bone marrow bank and kept frozen until injections in patients.

This study evaluated the use of BMCE via intraosseous or IV delivery, with a calcium phosphate scaffold, in irradiated bone reconstruction. The TBM-BCP mixture remains the gold standard for new bone formation without BMCE IV injections. However, IV injections of BMCE significantly enhance new bone formation when the defect is filled with TBM, with or without BCP, acting like an adjuvant to boost the bone formation.

BMCE opens new perspectives but raises many questions. Provided that these results are validated and the mechanism of action clarified, an application to humans may be considered.

## Supporting information

S1 Table Quantitative SEM study: Datas after semi-automatic image analyzer procedure. (XLSX)
