# Correction: Bone marrow cell extract promotes the regeneration of irradiated bone

**DOI:** 10.1371/journal.pone.0196145

**Published:** 2018-04-17

**Authors:** Guillaume Michel, Pauline Blery, Michaël Henoux, Jérôme Guicheux, Pierre Weiss, Emilie Dugast, Sophie Brouard, Olivier Malard, Florent Espitalier

Emilie Dugast should be included in the author byline. She should be listed as sixth author.

There are errors in the author affiliations. The affiliations should appear as shown here:

Guillaume Michel^1,2,3^, Pauline Blery^2,3,4^, Michaël Henoux^1,2,3^, Jérôme Guicheux^2,3^, Pierre Weiss^2,3,4^, Emilie Dugast^5,6^, Sophie Brouard^5,6^, Olivier Malard^1,2,3^, Florent Espitalier^1,2,3^

**1** Service d'O.R.L. et de chirurgie cervico-faciale, Centre Hospitalier Universitaire, Nantes, France, **2** INSERM, UMRS 791, LIOAD, Université de Nantes, Nantes, France, **3** PHU4 OTONN, Centre Hospitalier Universitaire, Nantes, France, **4** Service d'Odontologie Restauratrice et Chirurgicale, Centre Hospitalier Universitaire, Nantes, France, **5** INSERM UMR 1064, ITUN, Université de Nantes, Nantes, France, **6** Laboratoire d'immunologie (CIMNA), Centre Hospitalier Universitaire, Nantes, France

The correct citation is: Michel G, Blery P, Henoux M, Guicheux J, Weiss P, Dugast E, et al. (2017) Bone marrow cell extract promotes the regeneration of irradiated bone. PLoS ONE 12(5): e0178060. https://doi.org/10.1371/journal.pone.0178060
